# Do practical food groups work in an eating disorders day program? Long term outcomes of repeated exposure

**DOI:** 10.1186/2050-2974-2-S1-O23

**Published:** 2014-11-24

**Authors:** Jessica Wheatley, Susan Hart, Caitlin McMaster, Sarah Horsfield, Angela Thomas

**Affiliations:** 1 Derwent House Day Program, Sydney, Australia

## Background

Practical food groups expose patients to feared foods. This novel intervention is frequently undertaken in eating disorder treatment programs with tasks such as cooking or eating out. This intervention is clinically useful however there is little available evidence to support this approach. This study aimed to assess the effectiveness of practical food groups in a day program, and evaluated the long term outcomes.

## Method

Patients with mixed diagnoses attended practical food groups weekly as a standard part of treatment at Derwent House Day Program. A behavioural experiment format was adopted for the groups. 58 patients completed questionnaires at discharge, and data was collected at follow-up time points. Factors that impacted skill generalisability were recorded.

## Results

Mean BMI was 20 (BMI range 15-38), mean age was 25, and mean length of stay 7 weeks. 58 patients reported practical food groups as highly important at discharge, using a 5 point Likert scale (mean = 4.8). On follow-up 41 patients returned data, with similar ratings of importance.

## Conclusion

Overall, patients found practical food groups the most important intervention in day program treatment. In addition, skills acquired were largely generalised outside of treatment and maintained long term.

This abstract was presented in the **Service Initiatives** stream of the 2014 ANZAED Conference.

